# Quinazoline Derivatives as Potential Therapeutic Agents in Urinary Bladder Cancer Therapy

**DOI:** 10.3389/fchem.2021.765552

**Published:** 2021-11-03

**Authors:** Paulina Wdowiak, Joanna Matysiak, Piotr Kuszta, Katarzyna Czarnek, Ewa Niezabitowska, Tomasz Baj

**Affiliations:** ^1^ Department of Human Anatomy, Medical University of Lublin, Lublin, Poland; ^2^ Department of Chemistry, Faculty of Food Science and Biotechnology, University of Life Sciences in Lublin, Lublin, Poland; ^3^ Student Research Group at the Department of Human Anatomy, Medical University of Lublin, Lublin, Poland; ^4^ Institute of Health Sciences, The John Paul II Catholic University of Lublin, Lublin, Poland; ^5^ Department of Urology and Urological Oncology, Multidisciplinary Hospital in Lublin, Lublin, Poland; ^6^ Department of Pharmacognosy with the Medicinal Plant Garden, Medical University of Lublin, Lublin, Poland

**Keywords:** quinazoline derivatives, cancer treatment, urinary bladder cancer, cancer cell lines, anti-tumor activity

## Abstract

Cancer diseases remain major health problems in the world despite significant developments in diagnostic methods and medications. Many of the conventional therapies, however, have limitations due to multidrug resistance or severe side effects. Bladder cancer is a complex disorder, and can be classified according to its diverse genetic backgrounds and clinical features. A very promising direction in bladder cancer treatment is targeted therapy directed at specific molecular pathways. Derivatives of quinazolines constitute a large group of chemicals with a wide range of biological properties, and many quinazoline derivatives are approved for antitumor clinical use, e.g.,: erlotinib, gefitinib, afatinib, lapatinib, and vandetanib. The character of these depends mostly on the properties of the substituents and their presence and position on one of the cyclic compounds. Today, new quinazoline-based compounds are being designed and synthesized as potential drugs of anticancer potency against bladder cancers.

## 1 Introduction

### 1.1 Basic Cancer Concept and Statistics

Cancer is one of the diseases that claim the highest number of lives globally. According to WHO reports, cancerous diseases represent a severe burden for both men and women—over 244 million DALYs (Mattiuzzi and Lippi, 2019). The next two frequent disorders are ischemic heart disease (203 million DALYs) and cerebrovascular accidents (137 million DALYs). The number of cases of cancer is slightly higher in men. The WHO Global Cancer Observatory (GLOBOCAN) registry from 2020 showed that the top three most frequent cancers are breast, prostate and lung cancer. Moreover, the highest mortality is observed in lung, breast and colorectal cancer. Scientists are still searching for new methods of early detection and effective treatment of cancer.

Evaluation of a case of cancer is carried out considering the type of cancer present, risk assessment, and many other factors that have an influence on the treatment process. Regarding tumor biology, it is known that it involves an abnormal growth of cells. This growth has no functional purpose besides the ability to spread to adjacent structures and other parts of the organism. Some factors encourage the development of cancer, e.g., viruses, metals, and radiation damage (Czarnek et al., 2015; Czarnek et al., 2019). Genes that may underline tumor development and metastasis have also been extensively investigated.

Many of the advanced technologies that have been developed in recent years give researchers a better understanding of tumor biology and help find potential targets for effective treatment. Experiments using cancer cell lines or humanized mouse models provide a wide area for this search.

### 1.2 Bladder Cancer

Bladder cancer (BC) is a common genitourinary malignant tumor (Antoni et al., 2017). Initial symptoms are similar to those found in urinary tract infections—gross painless hematuria or urinary frequency. This fact can delay the appropriate, timely diagnosis and implementation of accurate treatment. According to the American Cancer Society (ACS), in 2021, an estimated 83, 000 adults in United States will be diagnosed with this type of cancer. The statistics show a decreasing tendency of new cases and deaths linked to bladder cancer in women. In men, the incidence rate has also been decreasing, but the mortality rate has remained unchanged. The risk of bladder cancer increases with age, with 9 out of 10 patients in United States being over 55 years of age (average age 73). It is the second most common urogenital malignancy, the first being prostate cancer (Bray et al., 2018).

Data has shown that the incidence of BC rises with age and is about three times higher in men than in women. However, women with urinary bladder cancer (UBC) have a poorer prognosis than men (Radkiewicz et al., 2020). Multiple risk factors of urothelial bladder cancer have been identified, one of the most important is cigarette smoking (Chavan et al., 2014). According to the ACS, almost half of all bladder cancer cases in both men and woman are caused by smoking. Another significant risk factor is exposure to certain industrial chemicals at work and in the general environment (according to International Agency for Research on Cancer). The most important are aromatic amines (e.g., benzidine and beta-naphthylamine using often as a dye). Other risk factors include dietary supplements (aristolochic acid), medicines (diabetes medicine pioglitazone), arsenic in water or a lack of clean potable water.

#### 1.2.1 Bladder Cancer Pathophysiology

The most common type of bladder cancer is urothelial carcinoma (also called transitional cell carcinoma). This cancer accounts for about 90% of all cases. Multifocality and recurrence are frequent in this subtype. Other subtypes, such as squamous cell carcinoma (5%), adenocarcinoma (2%), and mesenchymal carcinomas, are less common (Verma et al., 2012) and are associated with more advanced staging and higher mortality (Willis and Kamat, 2015). Generally, two kinds of bladder cancer, which differ in prognosis, can be distinguished: muscle-invasive (MIBC) and non-muscle-invasive (NMIBC) (Kates et al., 2017). Cancers affecting two layers of the bladder (NMIBC): urothelium and/or lamina propria represent about 70–85% of all cancer cases. MIBCs, invading the muscularis propria, usually are associated with a rapid progression. Also more commonly seen is metastasis to lymph nodes, bones, lungs, liver, and peritoneum (Shinagare et al., 2011). MIBCs tend to be more invasive, with a typically worse outcome following radiation, surgery and chemotherapy. NMIBCs are less aggressive, with a tendency towards recurrence. NMIBC tumors originate from cells of the inner layer of the bladder wall, the urothelium or transitional epithelium. Patients require surgical intervention and lifelong surveillance.

NMIBCs and MIBCs display different histopathological characteristics (Humphrey et al., 2016). The major determinant of carrying out a cystectomy is if the cancer has infiltrated the muscle layer. Urothelial bladder cancers (UBC) are mostly detected in older patients. The median age at diagnosis is 69 years in men and 73 in women. The 5-year relative survival rate for patients with urothelial bladder cancer (UBC) ranges from 97% (in early stage) to 22% (in more advanced cases) (Burger et al., 2013).

The TNM system identifies the stage and the grade of the bladder cancer. Accordingly: T signifies primary tumor, N—lymph node involvement, M—presence of metastasis (Amin et al., 2017). The T staging classification of bladder tumors, compiled by the American Joint Committee on Cancer (AJCC), is the most widely used staging system (Cornejo et al., 2020). This ranges from T0—no evidence of tumor, to T4a and T4b—where the tumor invades, respectively, the prostate, uterus, or vagina and the pelvic or abdominal wall. A high grade of the disease (poorly differentiated cells) corresponds with a more aggressive character of the lesion, with higher tendency to progression and dissemination.

Molecular studies about gene expression in urothelial cancers, designed by Lindgren et al., identified two intrinsic molecular types, MS1 and MS2 (Lindgren et al., 2012). These types mostly represent NMIBC and MIBC, respectively. Further profiling studies showed that primary bladder cancers can be divided into basal and luminal molecular types. Sjodahl et al. prepared gene-profiling experiments that subdivided the MS1 and MS2 classes into five additional molecular subclasses. The basis for this division were histological features and mutations presence in various genes: *PIK3CA, FGFR3* and *TP53.* These subtypes are: urobasal A (MS1a and MS1b), genomically unstable (MS2a subdivided into MS2a1 and MS2a2), urobasal B (MS2b2.1), squamous-cell carcinoma-like [SCC-like (MS2b2.2)] and a subtype highly infiltrated by non-tumor cells (MS2b1).

The character of basal tumors depends on the expression of markers of the basal layer of the urothelium (CD 44, cytokeratins: CK-5/6 and CK-14) (Sjödahl et al., 2012). Kamoun et al. demonstrated the occurrence of six molecular subtypes: luminal papillary (LumP), luminal nonspecified (LumNS), luminal unstable (LumU), stroma-rich, basal/squamous (Ba/Sq) and neuroendocrine-like (NE-like). Their data shows that, in bladder cancer, certain genes are particularly mutated: *CDKN2A, FGFR3,* proliferator-activated receptor gamma (PPARG), and human epidermal growth factor receptor 2 (HER2; *ERBB2*)*, TP53, E2F3* and *RB1* (Kamoun et al., 2020). The next step (ongoing) is to associate the molecular subtypes with clinical picture of the disease, ways of treatment and prognosis. A molecular analysis can help to choose the most appropriate disease-specific treatment.

#### 1.2.2 Bladder Cancer Therapy and Limitations

The basic methods of bladder cancer diagnosis are noninvasive imaging tests: CT, MRI and ultrasound, with cystoscopy as an invasive examination. The high accuracy of these tests is limited to advanced stages of BC. The gold standard remains biopsy. Noninvasive methods also include urine cytology, which is limited by poor sensitivity (Su et al., 2019). The FDA has also approved a few urinary tumor biomarkers, including bladder cancer antigen (BTA) or nuclear matrix protein 22 (NMP22) (Song et al., 2019). The development of new imaging technology (e.g., multi-slice spiral CT) and improved endoscopic techniques (fluorescence cystoscopy, optical coherence tomography or confocal laser endoscopy) enable the application of a greater number of diagnostic strategies that can help identify the disease in its early stages (Lalondrelle et al., 2012; Lerner and Goh, 2015; Lotan, 2015).

Patients with NMIBC undergo transurethral resection of tumor and appropriate chemotherapy or immunotherapy (Wein et al., 2016). The gold standard treatment for MIBC is radical cystectomy and pelvic lymphadenectomy, followed by chemotherapy. To enhance the immune response in patients with BC (NMIBC), immunotherapy is often applied. It also may help prevent tumor recurrence. The Bacillus Calmette-Guerin (BCG) vaccine has been used for bladder instillation after treatment of NMIBC (Kamat and Porten, 2014; Han et al., 2020), but the results are not satisfactory (Martínez-Piñeiro et al., 2015). The great limitation of this medication is the insensitivity of some patients to the BCG vaccine. Additionally, the high level of adverse drug reactions restricts its use in clinical applications, with most patients (80%) reporting cystitis-like symptoms. Dysuria, frequency of urination, and hematuria are the next most common adverse reactions (Harshman et al., 2015). The immunotherapy of BC also focuses on using dendritic cells to improve patient immunity and to enhance the elimination of tumor cells (Hurwitz et al., 2016). This kind of therapy is highly individual and requires the preparation of a unique vaccine in each case.

Targeted therapy is a promising and fast developing course in advanced medicine. This kind of treatment utilizes “specific” agents to recognize the target. These targets are particles present on the surface of tumor cells, e.g., cell surface molecules, membrane proteins or gene parts. As a result, the tumor cells should undergo apoptosis or necrosis. Targeted therapy usually has less adverse drug reactions and less nephro- or hepatotoxicity versus traditional chemotherapy. These advantages are demonstrated by therapy with monoclonal antibodies (MPDL3280A) that block the connection between PD-L1, PD-1 and CD80 (Powles et al., 2014). Associated with a worse prognosis, bladder cancer tumors often express high levels of PD ligand 1 (PD-L1) (McDermott and Atkins, 2013). Monoclonal antibody treatment has been approved by the FDA.

Another mAb that has been preliminarily proven highly safe is BCMab1. The target for this agent is AG-α3β1, a molecule expressed on bladder cancer cells that is responsible for crucial cell activities. Blocking this binding inhibits tumor cells growth, proliferation, invasiveness and metastasis (Li et al., 2014).

To develop targeted BC therapy, researchers should focus on finding factors that express high specificity and sensitivity with minimal adverse drug reactions. Research in the field of chemistry and pharmacology is still searching for more effective drugs with minimal side effects.

### 1.3 Quinazoline Derivatives

**FIGURE 1:**
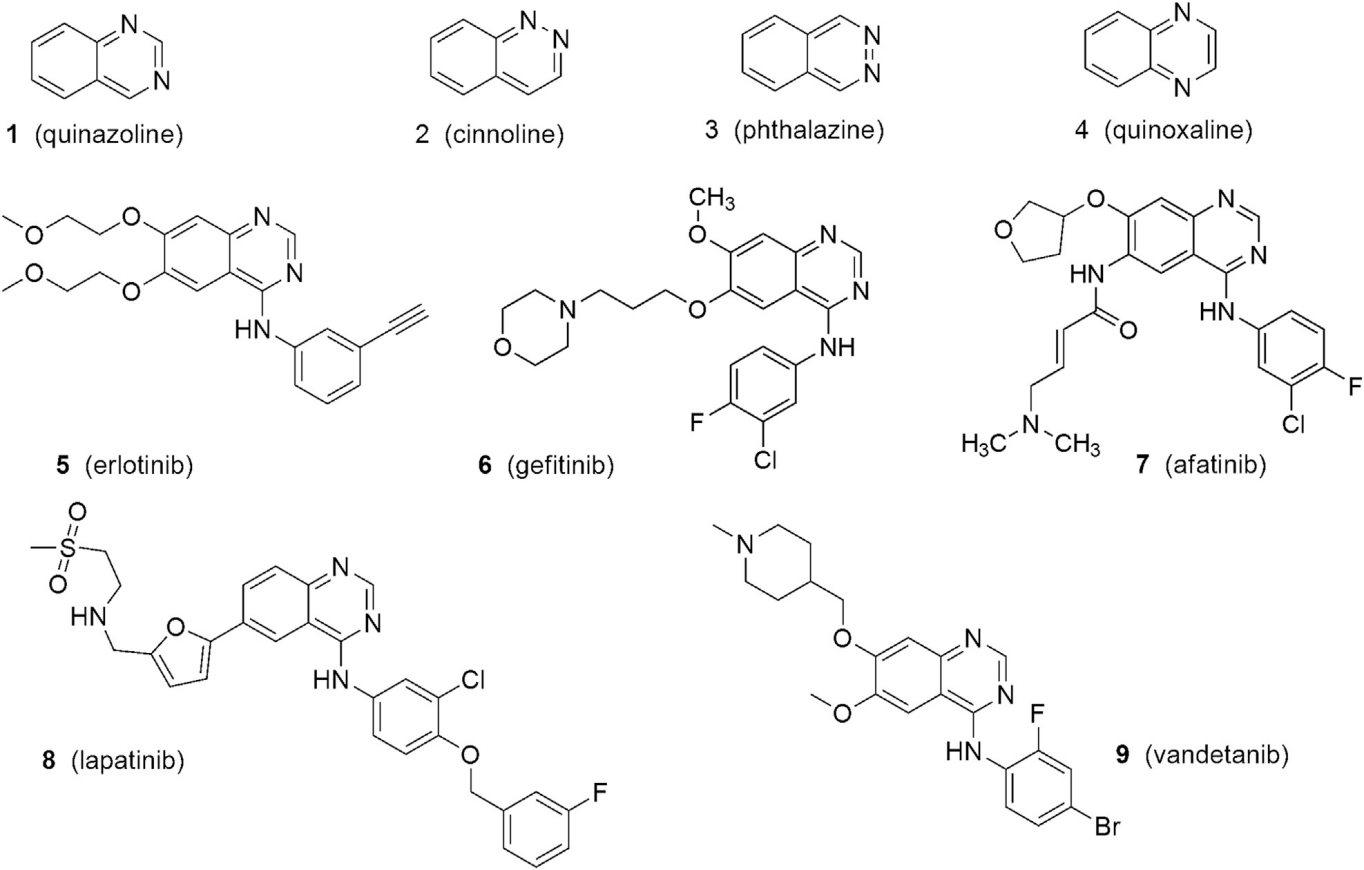
Structures of quinazoline and quinazoline-based anticancer drugs.

One of the most active classes of nitrogen containing heterocyclic aromatic compounds (Figure 1) (Mathew et al., 2017), quinazolines (1,3-benzodiazine) (Figure 1: 1) are a group of substances that display a wide range of activities. They belong to the benzodiazines—diazonaphthalenes, which contain two atoms of nitrogen in the same ring. Together with other isomers: cinnolines (1,2-benzodiazine) (Figure 1: 2), phthalazines (2,3-benzodiazine) (Figure 1: 3) and quinoxalines (1,4-benzodiazine) (Figure 1: 4), they have broad biological features and have important applications in medicine, pharmacy and agriculture (Mathew et al., 2017; Rakesh et al., 2017).

The structure of the main member of this group—quinazoline—contains two fused six-membered rings: a benzene ring combined with a pyrimidine ring. Quinazolinones, derivatives of quinazoline, are the oxidized form of quinazoline—ketoquinazoline (Devick, 2009). The location of the keto moiety is the base of classification: 2(1*H*)quinazolinones, 4(3*H*)quinazolinone and 2,4(1*H*,3*H*)quinazolinedione. These heterocyclic motifs often make up the essence of the structural skeleton in many drugs that express diverse properties and biological activities. The character of the quinazolines depends mostly on the properties of the substituents and their presence and position in one of the cyclic compounds (Mohamed et al., 2011).

Medically, derivatives of quinazoline are in common use. They can be chemically synthesized, as well as naturally obtained from various plant and animal species and microorganisms (Mohamed et al., 2011). Various methods are known for the synthesis of quinazolines. These substances are often applied in many kinds of therapies: anti-inflammatory, antiproliferative, analgesic, antidepressant, antibacterial, antifungal, and antiviral (Mohamed et al., 2012; Srivastav and Shantakumar, 2013). Quinazolines are also used in the treatment of tuberculosis, malaria, hypertension, convulsions, arrhythmia, and cancer diseases (Foster et al., 1999; Mohamed et al., 2012; Srivastav and Shantakumar, 2013). The scientific community is greatly interested in searching for new derivatives of quinazoline and discovering their properties in combating disease.

## 2 Antitumor Effects of Quinazoline Derivatives

Cancer is a disease that depends on changes in the cell cycle. This inevitably leads to uncontrolled cell division of abnormal cells. During the past few years, the Food and Drug Administration (FDA) has approved several innovative classes of anticancer chemotherapeutic factors from among the quinazoline derivatives. They have demonstrated significant therapeutic efficacy, especially against solid tumors. Many are approved for antitumor clinical use, e.g.,: erlotinib (*N*-(3-Ethynylphenyl)-6,7-bis(2- methoxyethoxy)quinazolin-4-amine) (Figure 1: 5), gefitinib (N-(3-chloro-4-fluorophenyl)-7-methoxy-6-(3-morpholin-4-ylpropoxy)quinazolin-4-amine) (Figure 1: 6), afatinib ((E)-N-[4-(3-chloro-4-fluoroanilino)-7-[(3S)-oxolan-3-yl]oxyquinazolin-6-yl]-4-(dimethylamino)but-2-enamide) (Figure 1: 7), lapatinib (N-[3-chloro-4-[(3-fluorophenyl)methoxy]phenyl]-6-[5-[(2-methylsulfonylethylamino)methyl]furan-2-yl]quinazolin-4-amine) (Figure 1: 8), vandetanib (N-(4-bromo-2-fluorophenyl)-6-methoxy-7-[(1-methylpiperidin-4-yl)methoxy]quinazolin-4-amine) (Figure 1: 9) (Bathula et al., 2020). The antitumor effects of quinazolines can manifest through numerous pathways.

### 2.1 Kinase Inhibition

**FIGURE 2:**
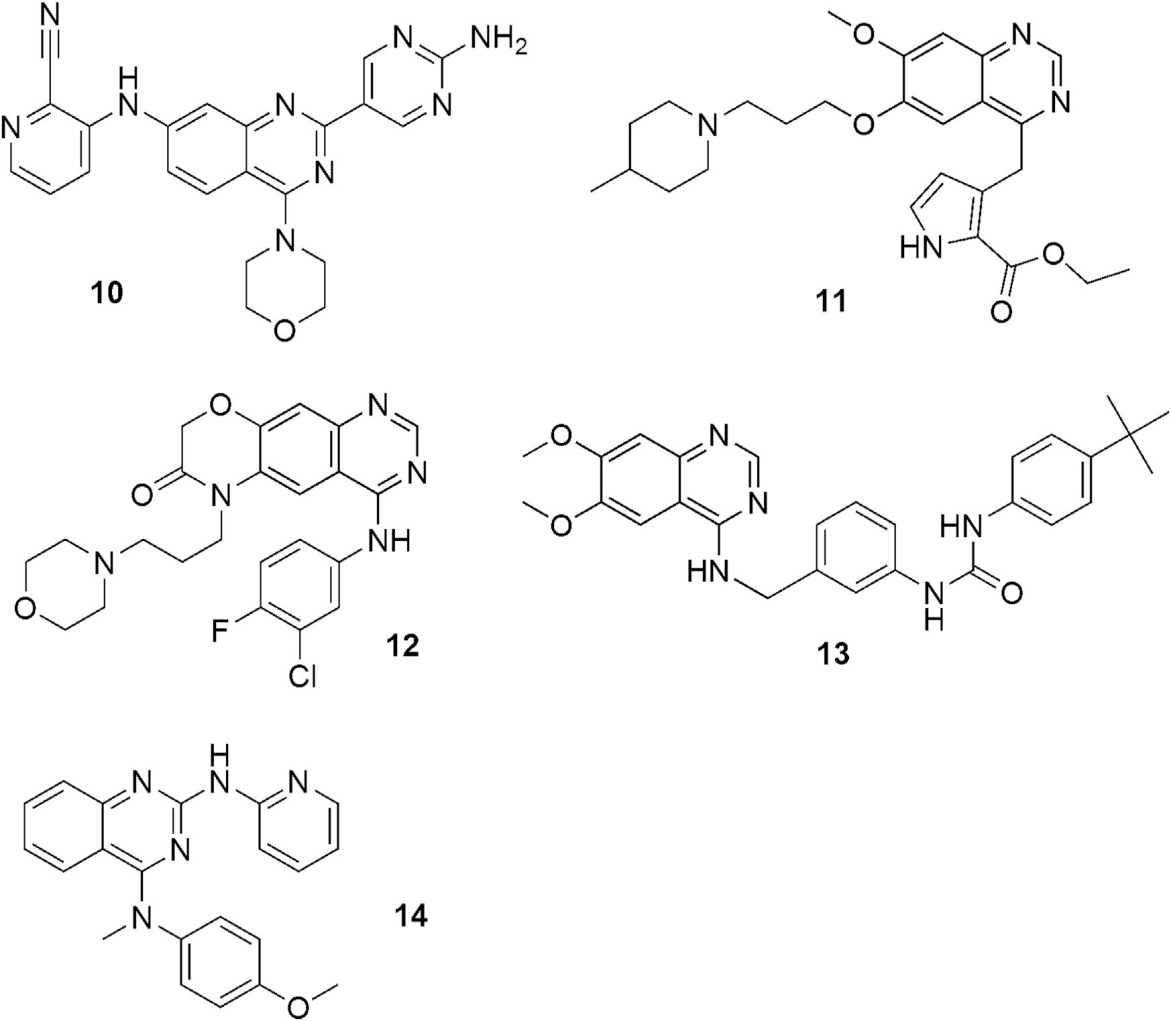
Structures of some compounds of antitumor potency incorporating quinazolinone frameworks.

An effective anticancer mechanism is the inhibition of phosphatidylinositol-3-kinase (PI3K) (with morpholine and nicotinonitrile moieties). This PI3K/Akt/mTOR pathway is crucial in regulatory functions in many cellular activities, such as cell growth, proliferation, differentiation and survival (Wullschleger et al., 2006; Peng et al., 2016a). Data has shown that many cancerous diseases develop through the activation of the PI3K/Akt/mTOR signaling network. Inhibition of the components of this network might, therefore, have utility in cancer treatment. The use of aminopyrimidinyl-4-morpholino-pyridinylquinazolin-7-amine derivatives as new effective PI3Kα inhibitors has been described (Figure 2: 10) (Peng et al., 2016b).

Other anticancer properties of quinazoline derivatives may be due to the inhibition of receptor tyrosine kinases (RKT) (e.g., epidermal growth factor receptor—EGFR) (Alqasoumi et al., 2010; Peng et al., 2016c). RTKs, which are involved in transmembrane signaling, are associated with the receptors of numerous growth factors (e.g., epidermal growth factor, vascular growth factor (Cadena and Gill, 1992). A cell response to a stimulus signal can have various cascading effects: cell proliferation, division, differentiation, hormone secretion or inhibition of any of these actions. Tyrosine kinases (Tks) and the process of phosphorylation are crucial for appropriate recognition of the aforementioned signal, its transduction into the cell and its amplification. The loss of control over these processes can develop into many dangerous disorders, as well as initiate carcinogenesis, accelerate cancer progression or metastasis (Porter and Vailancourt, 1998; Brunelleschi et al., 2002; Shagufta and Ahmad, 2017).

An important part of tumor biology research is an intense search for, and design of, antitumor drugs effective in inhibiting the activity of tyrosine kinases and which come with recommended physico-chemical properties that determine pharmacokinetic processes. The following are examples of tyrosine kinase inhibitors: pyrrole, morpholine, and urea moieties (Figure 2: 11–13) (Bhatia et al., 2020; Yang et al., 2021).

The first generation of EGFR inhibitors, e.g., erlotinib (Figure 1: 5) and gefitinib (Figure 1: 6), have applications in therapy of patients with non-small cell lung cancer (NSCLC) with EGFR mutations (Faehling et al., 2018; Zhang et al., 2018) and have a reversible mechanism of action. The second generation of inhibitors, including afatinib (Figure 1: 7), with irreversible inhibitors of mutant EGFR, also express high efficacy against NSCLC (Sharma and Graziano, 2018). Despite these successes in treatment, it emerged that patients can develop tumor resistance against the activity of these targeted drugs. This comes about via the mutations in the tyrosine kinase domain of EGFR, or the activation of other signaling pathways in cancer cells which take on the role of the suppressed pathways.

The third generation of kinase inhibitors involve the inhibition of the VEGF (vascular endothelial growth factor) receptor—the stimulation of which initiates signal cascades that induce angiogenesis. VEGFRs are up-regulated in tumors, as these have a high metabolic requirement for oxygen. Vandetanib (Figure 1: 9), a quinazoline derivative, belongs to this generation. This shows promising results in medullary thyroid cancer treatment (Fallahi et al., 2016).

### 2.2 Tubulin Inhibition

Quinazolines can also exhibit apoptotic properties in cancerous cell lines (Li et al., 2019) by means of acting as tubulin inhibitors. Tubulin is the structural protein of microtubules and plays a crucial role in cell division. If the drug binds to tubulin, it prevents the formation of the microtubules required for the formation of the mitotic spindle. This property can lead to an antimetastatic action because of inhibition of cell migration and invasiveness (Xia et al., 2020). Vincristine and vinblastine are examples of known drugs used in oncology for this purpose. Vinblastine is used in the treatment of Hodgkin’s disease, a form of lymphoid cancer, and vincristine is used clinically in combating children’s leukemia (Lönnerholm et al., 2008). A series of new effective inhibitors from the group of 2,4-diamino-N,N-disubstituted quinazolines has been described as being effective anti-cancer compounds (Figure 2: 14) (Li et al., 2019).

### 2.3 Diverse Antitumor Mechanism of Hybrid Compounds

**FIGURE 3:**
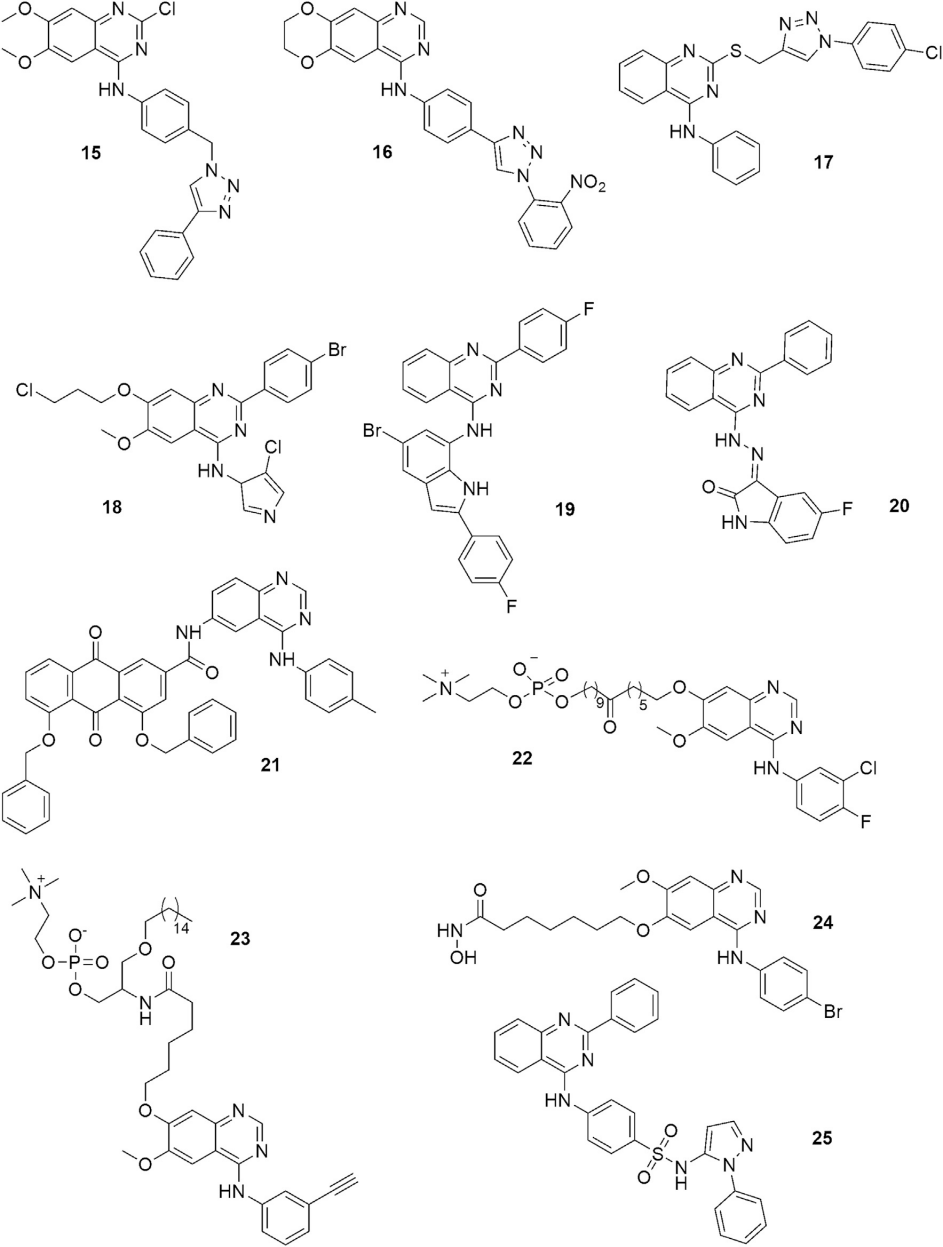
Structures of quinazoline-based hybrid compounds.

Recent investigations indicate that the quinazoline derivatives, upon being administered as components of multifunctional hybrid compounds, can significantly increase the effectiveness of antitumor treatment (Prashant et al., 2020). The quinazoline ring has been attached to various heterocyclic systems using linkers of different lengths and electronic properties. Linear pharmacophores have also sometimes used (Auti et al., 2020). One of the important heterocyclic scaffolds used in medical chemistry is the 1,2,3-triazole ring, also often explored in the design of quinazoline anti-tumor hybrids. A small set of phenyltriazole and 2-amioquinazoline conjugates was described by Banerji et al. (2018). The compounds exhibited antiproliferative potency against some human cancer cell lines, especially against MCF-4. The most active analog (Figure 3:15) decreased the expression of EGFR, p-EGFR and induced apoptosis through reactive oxygen species generation. The direct EGFR inhibition or that of EGFR function *via* the excessive ROS generation or both could be a viable cancer treatment approach.

Other phenyltriazole and 2-amioquinazoline hybrids were described by Giang et al. (2018), and in research, were found to be active against the KB, HepG2, and SK-Lu-1 human cancer cell lines. The most potent derivative (Figure 3: 16), with a nitrophenyl substituent and a rigid cyclic system -OCH_2_CH_2_- on the quinazoline ring, exhibited 100-fold higher cytotoxicity, in comparison with erlotinib. Docking studies suggest that such compounds may bind with the EGFR tyrosine kinase domains.

Several researchers have designed and synthesized hybrid compounds where a triazole ring was linked through the thioether linkage (Figure 3: 17) (Song et al., 2017). Some compounds were found to exhibit good growth inhibition against the human HGC-27, MGC-803, EC-109, and MCF7 cells. The most potent analog (Figure 2: 20), showed cytotoxicity below 1 μM (MF-7, IC_50_), and was more effective than 5-fluorouracyl.

Recently attempts have been made to synthetize a compound using a pyrrole ring. The obtained 2-substituted 4-anilinoquinazoline-pyrrrole conjugates (Figure 3: 18) showed moderate antiproliferative potency against the MCF-7 and A-549 cells (Bathula et al., 2020).

There are also known hybrids of quinazoline and indole with great anti-cancer potential. The studies of a series of 2-aryl-4-chloroquinazoline conjugates with the 7-amino-2-aryl-5-bromoindoles have demonstrated that the derivatives are characterized by antiproliferative action against the Caco-2, C3A, MCF-7, and HeLa cells. The most active derivative (Figure 3: 19) induced apoptosis in the Caco-2 and C3A cells and showed a significant inhibitory activity towards GFR (IC_50_ = 40.7 nM). Molecular docking studies have revealed that the compound could bind to the ATP region of EGFR, similarly to erlotinib (Mphahlele et al., 2018).

In another study, the indole system, isatin moiety, was attached by means of hydrazone linkage (Fares et al., 2015). The most active compound (Figure 3: 20) demonstrated good potency, with IC_50_ values ranging from 1.0 to 14.3 μM against MCF-7, HepG2 and HT-29 cancer cell lines. The anticancer potency of such derivatives is due to the activation of the mitochondrial apoptotic pathway. The compounds induced the activation of caspase-3 and reduced expression of the anti-apoptotic protein Bcl-2 in the HepG2 cells.

Novel hybrid analogues generated by a combination of quinazolinone and allylphenyl quinoxaline can significantly increase anti-cancer activity against a few cancer cell lines: HeLa, 161 MIAPaCa, MDA-MB-231, and IMR32 (Palem et al., 2016). Docking studies suggest that these compounds could act as promising DNA intercalates.

A further two ring-fused system connected with the aminoquinazoline scaffold is benzimidazole. The benzimidazole ring may be responsible for targeting Aurora kinase and may inhibit proliferation of cancer cells. In one study, a small set of conjugates was evaluated on a very wide panel of 60 human cancer cell lines. The most active analogs at the concentration of 10 μM showed a 90% growth inhibition of cancer cells (Sharma et al., 2013). The most active conjugates developed by Luxami and co-workers showed Aurora kinase inhibition, with IC_50_ = 0.53 mM (Luxami et al., 2015).

Other researchers studied purine-quinazoline hybrids (Kapadiya and Khunt, 2019). Identified by a cytotoxic study against NCI-60 cell-lines, its anticancer potency was about 7 µM (GI_50_) for the most active compounds.

Mitoxantrone is a classical anthraquinone-based antineoplastic drug. Its mechanism of action involves DNA intercalation and induction of DNA damage (Atwal et al., 2017). Other anthraquinone derivatives (GXHSWAQ-1) enhance sensitivity of some cancer cells to radiotherapy through targeting (Mo et al., 2014). Recently, certain novel anthraquinone−quinazoline multitarget hybrids were designed, synthesized and evaluated. The most potent of these compounds (Figure 3: 21) significantly downregulated the expression of p-EGFR protein, promoted the rearrangement of F-actin filaments and destruction of cytoskeleton, induced DNA damage and a remarkable apoptosis effect, and enhanced radiosensitivity of the A549 cells. Some compounds showed significant antiproliferation activity against human cancer cell lines. As multitargeted ligands, they can be consided to be good anticancer drug candidates (Liang et al., 2020).

Novel hybrids were generated as multitarget anticancer agents by the combination of quinazoline-based anti-cancer drugs and alkylphospholipids. In research, some alkylphospholipids were discovered to exhibit anticancer potency and act as Akt phosphorylation inhibitors. They were noted to interfere with lipids metabolism and lipids-dependent signaling cascades (van Blitterswijk and Verheij, 2013). Alam and co-workers developed several alkylphosphocholine-gefitinib conjugates (Figure 3: 22). These compounds showed cytotoxic properties against MCF-7, A431, HepG2 and A549 cells. Their cytotoxic effect was weaker than that of the parent gefitinib, but comparable to erlotinib and miltefosine. Moreover, the derived compounds showed strong inhibition of EGFR kinase, but were not effective inhibitors of Akt phosphorylation (Alam et al., 2018).

Hybridization of erlotinib and alkylphospholipids gave the compounds promising broad-spectrum cytotoxic activity. Herein, the most active compound (Figure 3: 23) showed higher efficacy than the reference erlotinib and miltefosine. Statistical correlation analysis indicates that both mechanisms (EGFR kinase and Akt phosphorylation inhibition) have an influence on the cytotoxic effect of compounds towards human cancer cells (Alam et al., 2019).

Incorporating the attributes of a bifunctional molecule that acts as an inhibitor of both of PI3K/AKT and GFR pathways could be a useful approach in designing novel anticancer drugs. To obtain duals inhibitors, Peng and co-workers took 4-aminoquinazoline—vandetanib pharmacophore and hydroxamic acid as the HDAC inhibitor for the hybridization (Figure 3: 24). The derivatives exhibited cytotoxic activity against the human cancer cell line and inhibited some proteins involved in cancer growth. Furthermore, the most active compound showed a significant inhibitory potency against VEGRF-2 (IC_50_ = 74 nM) and HDAC (IC_50_ = 2.2 nM), while its cytotoxic activity against MCF-7 was found to be equal to 0.85 µM (IC_50_). As follows from the docking studies, the compound binds at the active site of histone deacetylase-like protein (HDLP) and VEGFR-2 kinase (Peng et al., 2016a).

In the study of Ghorab et al., quinazoline-sulfonamide hybrids demonstrated minor cytotoxic activity against the human A549, HeLa, LoVo and MDA-MB-231 cancer cell lines (Figure 3: 25) (Ghorab et al., 2016). Sulfonamides are known to be anticancer agents that act through the following mechanisms: carbonic anhydrase (CA) inhibition, the disruption of microtubule assembly and cell cycle perturbation (Sławiński et al., 2013; Auti et al., 2020).

## 3 Quinazoline Derivatives in Urinary Bladder Cancer Therapy

### 3.1 Quinazoline Based Drugs as Epidermal Growth Factor Receptor Inhibitors

One relatively new approach in the treatment of bladder cancer is targeted therapy directed at specific molecular pathways. The high diversity of bladder cancer is the main difficulty in selecting an appropriate target for drugs. Neoplastic cells have tendency to “escape” and change one pathway to another one for the purposes of growth. One of the potential therapeutic targets is the epidermal growth factor receptor (EGFR). In bladder cancer, overexpression of EGFR on the luminal surface is frequently seen and it correlates with higher tumor staging and a poorer prognosis (Neal et al., 1990; Bryan et al., 2015). Gefitinib is an EGFR tyrosine kinase inhibitor (Figure 1: 6).

Peng et al. conducted an experiment in which three different bladder cancer cell lines were treated with gefitinib alone, or in combination with metformin (Peng et al., 2016b). Metformin is a well-known and widely used anti-diabetic drug. It also exhibits antitumor properties confirmed *in vitro* and *in vivo.* The combination of metformin with gefitinib was found to inhibit bladder cancer proliferation.

The projected mechanism of action was seen to be dependent on metabolic control and influence on the EGFR signaling pathway. Results indicated significantly higher inhibition of BC cell proliferation after administering the combination of metformin and gefitinib. Colony formation was inhibited as well. This synergistic inhibitory effect was confirmed through an apoptosis assay. The applied combination interfered with tumor cell metabolism, inhibited the EGFR signaling pathway and activated the AMPK signaling pathway (AMP-activated protein kinase).

In further studies, researchers examined another drug combination: phenformin and gefitinib (Huang et al., 2018). As a derivative of metformin, phenformin displayed even better anticancer potency at lower doses (Wang et al., 2015). This experiment was based on one mouse, and two human bladder cancer cell lines, respectively, MB49, T24, and UMUC3. The data obtained showed promising results regarding inhibition of bladder cancer proliferation, colony formation and migration. The effective concentration of phenformin turned out to be tenfold lower than metformin. This aspect is crucial in light of low toxicity and potential clinical trials on patients.

Another experiment designed by Sakai et al*.* focused on lapatinib—a tyrosine kinase inhibitor belonging to the 4-anilinoquinazoline class (Figure 1: 8) (Sakai et al., 2018). The target for this drug is the kinase domain of HER2 and EGFR (Wood et al., 2004). They used a canine TCC cell line (urothelial carcinoma, transitional cell carcinoma). The data obtained revealed that lapatinib brought about an inhibition of the phosphorylation of HER2 and EGFR that was mostly mediated by inhibition of the HER2-MAPK/Erk pathway (Ciardiello et al., 2000). Lapatinib also provoked an anti-tumour effect in the canine TCC-engrafted mouse model with minimal side effects. This research is very promising, especially considering that the anti-tumor activity of lapatinib was also observed in human bladder cancer cell lines (McHugh et al., 2009), wherein it exhibited overexpression of HER2 determined by an immunochemistry range from 8% up to 80% (Jimenez et al., 2001; Powles et al., 2017). Unfortunately, treatment of human bladder cancer patients using lapatinib (Figure 1: 8) during a stage III randomized trial was unsuccessful (Powles et al., 2017).

A different animal-model experiment demonstrated that intermittent administration of erlotinib (EGFR inhibitor) (Figure 1: 5) in combination with naproxen provides significant efficacy data in OH-BBN-induced rat bladder tumors (histologically similar to human transitional cell carcinoma) (Lubet et al., 2010; Mohammed et al., 2020). This treatment exhibited no serious side-effects, while demonstrating nominal toxicity.

### 3.2 Quinazoline-Based Antihypertensive Drugs

**FIGURE 4:**
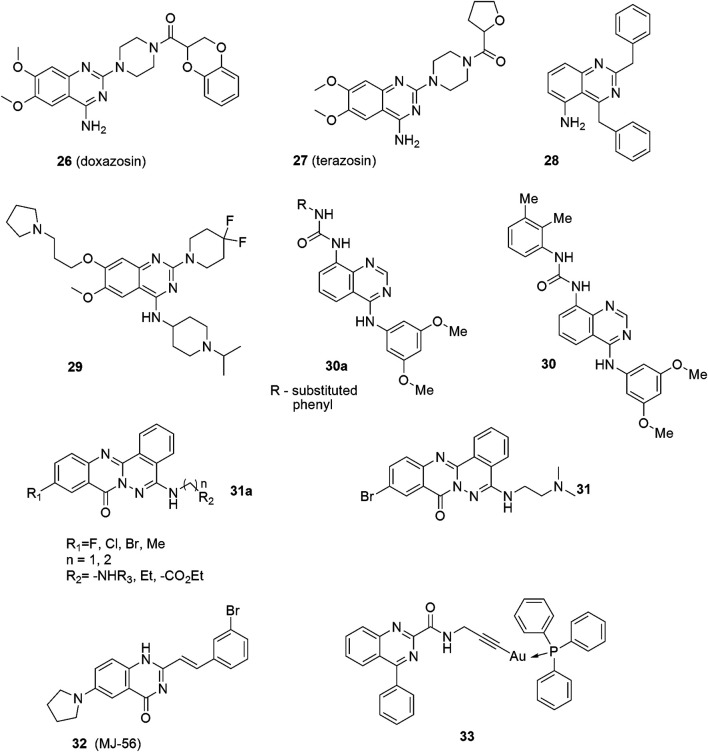
Structures of quinazoline derivatives exhibiting anticancer effects against urinary bladder cancers.

The other quinazoline-based drugs also tested as human bladder cancer anticancer agents are Doxazosin (Figure 4: 26) and terazosin (Figure 4: 27), both α1-adrenoreceptor antagonists. These are FDA-approved drugs for the treatment of systemic hypertension and benign prostatic hypertrophy (BPH). They exhibit a few well-tolerated side effects, mostly dizziness. Reduction in the risk of developing bladder cancer in men treated with the quinazoline-based α1-adrenoceptor antagonists was observed (Walden et al., 1997; Kyprianou et al., 2009). Other research revealed that human bladder cancer cells are sensitive to the apoptotic effects of terazosin and that bladder tumors exhibit reduced tissue vascularity (Tahmatzopoulos et al., 2005; Martin et al., 2008). Recently, the above results have been confirmed by Mihalopoulos and coworkers (Mihalopoulos et al., 2020).

### 3.3 Aminoquinazoline Derivatives of Antiproliferative Potency Against Urinary Bladder Cancer Cells

Several new quinazoline-based compounds have been designed and synthesized as potential therapies against bladder cancers. The compound, 2,4-Dibenzylaminoquinazoline (Figure 4: 28), for example, exhibits cytostatic and apoptotic effects against bladder cancer cells, mainly in a transcription independent manner (Cubedo et al., 2006). Cao and co-workers described fluorinated diperidinylpyrrolidinylquinazolin amine UNC0642 (Figure 4: 29) as an UBC agent acting under both *in vitro* and *in vivo* conditions (Cao et al., 2019). The compound decreased the viability of J82, 5637, and T24 cells, as well as reduced the levels of histone H3K9me2 and the downstream target of G9a, hence, increasing apoptosis. The anticancer effect was confirmed in the *in vivo* mouse model. Analysis of the clinical databases shows that G9a is significantly overexpressed in UBC patients, and, therefore, it can be a promising therapeutic target for future UBC drug design (Casciello et al., 2015).

Arylaminoquinazolinylurea derivatives (Figure 4: 30a—general formula) were described as anticancer agents by Kim et al. (2016). Their research involving structure-activity elucidation showed that compounds with electron-donating substituents were generally more potent than those possessing electron-withdrawing attributes, except for derivatives with a–NO_2_ substituent. Among these, the dimethylphenylurea derivatives exhibited the highest potency. The most active analog with the 3,4-dimethylphenyl substituent (Figure 4: 30) exhibited *in vitro* antiproliferative activities against the RT112 bladder cancer cell line in the small nanomolar range (IG_50_) and was more potent than the reference drug. In contrast, analogs with fluorinated and chlorinated phenyl moiety displayed a poor potency. Moreover, replacing the urea system with an amide group produced compounds of very low activity. The most potent compound 30 also acted as a weak FGFR3 kinase inhibitor.

### 3.4 Quinazolinone Derivatives of Antiproliferative Potency Against Urinary Bladder Cancer Cells

Zhang et. al designed and synthesized a group of substances based on the phthalazino[1,2-b]quinazolinone motif (Figure 4: 31a—general formula) (Zhang et al., 2017). The structure-activity analysis showed that replacing the hydrogen atom at the 10-position of the phthalazino[1,2-*b*]quinazolin-8-one skeleton with a halogen atom or a methyl substituent increases the cytotoxicity. Among the synthesized compounds, the Br derivatives were found to be the most potent. The substituent in the 5-position of the phthalazino[1,2-*b*]quinazolin-8-one skeleton also had a very important effect on potency. In general, the aminoalkylamine side chains gave a better antiproliferative activity than did 4-methyl-*N*-piperazinyl. In the study, compounds with *N*-morpholinyl, alkyl, or ω-alkoxycarbonyl substituted alkyl almost lost the cytotoxicity against T24 cells.

In the work of Zhang et al., the tail of the aminoalkylamine side chain was found to also significantly affect the potency of the compounds. Therein, derivatives with a dimethylamino at the tail were noted to be more potent than those with diethylamino or diiospropylamino moieties. When the tail of the aminoalkylamine was replaced by the *N*-morpholinyl, the cytotoxicity of the compounds was reduced. Furthermore, a link of aminoethyl was discovered to be more favourable than that of an aminopropyl. The proliferation of T24 cancer cells was most effectively inhibited by the compound with the dimethylamino moiety at the tail (Figure 4: 31). It acted as a p53 activator in bladder cancer cells and showed promising anticancer activities. This antitumor effect is likely dependent on the induction of apoptosis based on mitochondria-targeted accumulation of phospho-p53.

Different strategies and approaches utilizing compounds of this type have also been applied to obtain better efficacy and safety. A novel quinazoline derivative, MJ-56 (Figure 4: 32) was described as a phototoxic agent against human bladder cancer cells (Chen et al., 2018). It emits green fluorescence in the cytosol, and exhibits phototoxicity against T24 cells with minimal influence on the uroepithelial cells.

### 3.5 Metal Complexes With Quinazoline Derivatives

Recently, gold(I) complexes with a quinazoline carboxamide alkynyl ligand have been described as effective cytotoxic agents against bladder cancer cells (Tabrizi et al., 2021). The docking studies indicated that the free ligand (Figure 4: 33) interacts with the translocator protein 18 kDa (TSPO) which plays a crucial role in mitochondrial biochemistry, quality control, transporting of heme precursors, immunomodulation, the regulation of the energy metabolism, steroidogenesis and cell proliferation. TSPO is overexpressed in many cancer types and its level correlates with tumor malignancy, as well as cancer progression.

## 4 Discussion

Cancer is a disease caused by abnormalities in the cell cycle, resulting in uncontrolled cell division of the changed cells. Cancerous diseases are, without doubt, one of the major health problems worldwide. There are many anti-cancer drugs and therapies utilized in modern medicine, but there is still the need to seek new ones, because of the problems of side effects and drug resistance. Quinazolines and their derivatives are a large group of chemical compounds that definitively play a key role in the area of pharmacochemistry. The biological and pharmacological properties of quinazolines primarily depend on their structure. However, high chemical reactivity hampers experiments regarding their particular anti-tumor activity and metastatic inhibition, and various quinazoline derivatives exhibit insufficient and poor solubility. Despite that, this group of chemicals is still a promising base for synthesizing new anticancer agents that can potentially target specific receptors.

Quinazoline and quinazolinone derivatives are a very diverse group and almost each of them expresses strong and frequently different biological properties (Asif, 2014), and this is the reason why many centers are involved in searching for new possibilities of using them as novel cancer therapies. It is a very promising direction in drug design studies, especially in the research area of various malignancies.

Tumorogenesis is a complicated multi-step process, and tumor cells often undergo metabolic processes that change their one ‘style’ of growth (Vallo et al., 2015). Huang et.al showed that application of two biologically different agents may be a successful therapeutic strategy in cancer. For example, gefitinib activates the AMPK signaling pathway and phenformin inhibits EGFR signaling in a dose-dependent manner. This combination brings together both inhibition of bladder cancer cell proliferation and colony formation, and stimulation of apoptosis in those same cells. Furthermore, tumour microenvironments have been strongly considered as one of the crucial factors necessary not only for tumour growth and progression, but for drug response (Hu and Polyak, 2008), especially in urogenital malignancies with specific diverse environment.

Application of targeted drugs during various therapies has brought about great success in treating some cancers. However, often, this treatment is assisted by immunosuppressive agents or intravesical chemotherapy, which can generate additional handicaps such as varying degrees of side effects, for instance, bone marrow suppression (Gontero et al., 2010). In the treatment of bladder cancer, there is a great requirement to develop a successful pathway of administration. Over all, research indicates that quinazoline derivatives could be potentially effective therapeutic agents in urinary bladder cancer therapy.
